# Feature Extraction and Automatic Recognition Model Construction for Head Back Posture During the Parturition Process in Dairy Cows

**DOI:** 10.3390/ani15172470

**Published:** 2025-08-22

**Authors:** Xia Li, Yifeng Song, Xiaoping An, Zhalaga, Yuning An, Yuan Wang, Na Liu, Jiaxu Gu, Jingwei Qi

**Affiliations:** 1 College of Animal Science, Inner Mongolia Agricultural University, Hohhot 010018, China; lixia2270@126.com (X.L.); songyifeng777@163.com (Y.S.); xiaoping_an@163.com (X.A.); 15124748201@163.com (Z.); anyuning@emails.imau.edu.cn (Y.A.); wangyuan@imau.edu.cn (Y.W.); liuna1988@imau.edu.cn (N.L.); gujiaxu613623@163.com (J.G.); 2 National Center of Technology Innovation for Dairy-Breeding and Production Research Subcenter, Hohhot 010018, China; 3 Key Laboratory of Smart Animal Husbandry at Universities of Inner Mongolia Autonomous Region, Integrated Research Platform of Smart Animal Husbandry at Universities of Inner Mongolia, Inner Mongolia Herbivorous Livestock Feed Engineering Technology Research Center, Hohhot 010018, China

**Keywords:** parturition process, dairy cows, computer vision, YOLOv8

## Abstract

The ‘head back’ posture is a pronounced and significant behavioral trait during bovine parturition, commonly interpreted as a natural response to the pain associated with parturition. Leveraging computer vision technology for real-time monitoring of parturition behaviors can provide timely assistance during calving and enhance animal welfare. This study initially evaluated the head back posture in cows of different types, finding that primiparous cows and those delivering calves weighing over 43 kg exhibited prolonged durations of both labor and head back posture. A model was developed using the YOLOv8 algorithm with 25,617 images to recognize and classify changes in head posture during parturition, including positions like lying with or without head back. The model demonstrated robust predictive performance with a precision (*P*) of 69.76%, recall (*R*) of 75.35%, average precision (*AP*) of 70.12%, and *F*_1_ score of 0.71. Furthermore, the model’s capability to recognize postures from different camera angles and under varying environmental conditions was assessed. Notably, images captured from an abdominal angle achieved *AP* exceeding 90%, with consistent stability under varying lighting conditions, including sunny and overcast weather, during both daytime and nighttime. Behavioral analysis showed that the parturition duration and total duration of head back posture in primiparous cows were significantly higher than those in multiparous cows (*p* < 0.05), and the changing trends of motor performance between primiparous and multiparous cows were consistent across different parturition stages. Additionally, the correlation between calf birth weight and maternal behavior was stronger in primiparous cows than in multiparous cows, further indicating obvious differences in physiological and behavioral responses of cows during primiparous and multiparous parturition. This study underscores the potential of computer vision applications in enhancing real-time intervention and promoting welfare during bovine parturition.

## Simple Summary

The “head back” behavior of dairy cows during calving is an important signal of parturition pain, but traditional monitoring methods struggle to assess it in real time. This study used computer vision technology to develop a non-contact monitoring model capable of recognizing calving postures by analyzing over 25,000 images. The results showed that first-calving cows (primiparous) had longer labor durations and more prolonged head back posture. Cows giving birth to calves heavier than 43 kg also exhibited these trends. The model performed stably under different camera angles and lighting conditions, achieving over 90% accuracy when capturing images from an abdominal perspective. The study confirmed significant differences in calving behaviors between primiparous and multiparous cows, with calf birth weight notably influencing maternal behavior. This technology enables real-time detection of abnormal calving, supporting precise farm management and contributing to improved cow welfare and more efficient calving intervention.

## 1. Introduction

Parturition, a critical phase in the dairy cow’s production cycle, encompasses three distinct stages: cervical dilation, expulsion of the fetus, and delivery of the placenta. This process is typically accompanied by acute pain, with complications at any stage potentially leading to dystocia, which adversely affects both the cow and the calf [1]. During parturition, dairy cows exhibit various behavioral and physiological changes, such as abnormal lying patterns, altered rumination duration, changes in step count, tail lifting, and fluctuations in body temperature and hormonal levels [2,3,4,5]. Alterations in head posture during parturition not only reflect the animal’s pain and stress levels but also potentially influence the efficiency and safety of the calving process. This posture is considered a natural response to the pain of parturition, likely triggered by increased abdominal pressure and uterine contractions [6]. During contractions, cows adjust their body posture by extending their heads backward to alleviate discomfort and adapt to the physiological changes occurring throughout parturition. The emergence of the head back behavior may serve as an early indicator of imminent parturition. Monitoring this behavior can assist farm personnel in timely identification of cows about to give birth, facilitating the provision of necessary care and supervision [7,8]. Because of its distinctive appearance and strong predictive value for calving events, effective monitoring and management of the head back posture are crucial for optimizing parturition management and reducing the risk of complications.

Despite the significance of parturition for both the cow and the neonate, calving monitoring and assistance are often neglected and remain weak links globally. Traditional behavioral monitoring relies heavily on subjective human observation, making it time consuming, labor intensive, and susceptible to inter-observer variability [7]. Additionally, prolonged labor or dystocia and inappropriate timing of assistance (either late or premature) can impair maternal welfare, fertility, and milk production, as well as the survival, growth, and future performance of the calf [9,10]. In recent years, rapid advancements in precision livestock farming have led to the development of a variety of smart sensors—including accelerometers, rumination collars, temperature loggers, and GPS-based activity trackers—that enable continuous and automated monitoring of physiological and behavioral parameters in dairy cattle [11,12,13]. Among these technologies, wearable collar and ear tag devices are now widely adopted on commercial farms for real-time tracking of activity patterns, estrus detection, health status, and especially rumination time—a principal parameter closely associated with parturition status, metabolic health, reproductive performance, and welfare assessment. Furthermore, breakthroughs in computer vision technology have facilitated non-invasive video-based monitoring approaches capable of recognizing complex postural changes and behavioral events without direct animal contact [14,15,16,17,18,19]. These technologies have been successfully applied in various contexts, including monitoring of calving behaviors [20], automated behavioral monitoring (e.g., activity patterns and postural transitions) [14], and prediction of calving time [21]. However, their application in monitoring specific behaviors, such as head back posture, remains largely unexplored.

Our study initially utilized computer vision technology to analyze the duration of various postures—head back, no head back, and other postures—in primiparous and multiparous cows, as well as in cows with different calf birth weights. We then developed a model employing the YOLOv8 algorithm, which automatically recognizes and categorizes changes in head posture during parturition. The model demonstrates a satisfactory capability in identifying head back posture, providing a data-driven foundation and technological support for intelligent calving management in modern dairy farming.

## 2. Materials and Methods

### 2.1. Experimental Design and Video Capture

The study was conducted at a large dairy farm in Hohhot (Hohhot, China, 40°48′ N, 111°39′ E). This study employs the U.S. five-point scoring system to evaluate dairy cows’ body condition. Given that physical conditions may cause deviations of 0.5–0.75, the Body Condition Score (BCS) is categorized into intervals of 0.25, resulting in five levels: 3.25, 3.5, 3.75, 4.0, and 4.25. We specifically selected cows with BCS scores between 3.25 and 3.5 under standardized management conditions for this research. Video capture was carried out from April to September 2023, documenting the parturition of Holstein dairy cows during various weather conditions, including daytime, nighttime, overcast, and sunny periods. For model development, a total of 569 cows were included, comprising two groups, 78 calving cows and 491 non-calving cows, which were distributed across four separate barns. For the comparative analysis of head postures, cows were further divided into groups based on parity (primiparous [*n* = 20] and multiparous [*n* = 20]) and calf birth weight (low birth weight < 37 kg [*n* = 20] and high birth weight > 43 kg [*n* = 20]).

**Figure 1:**
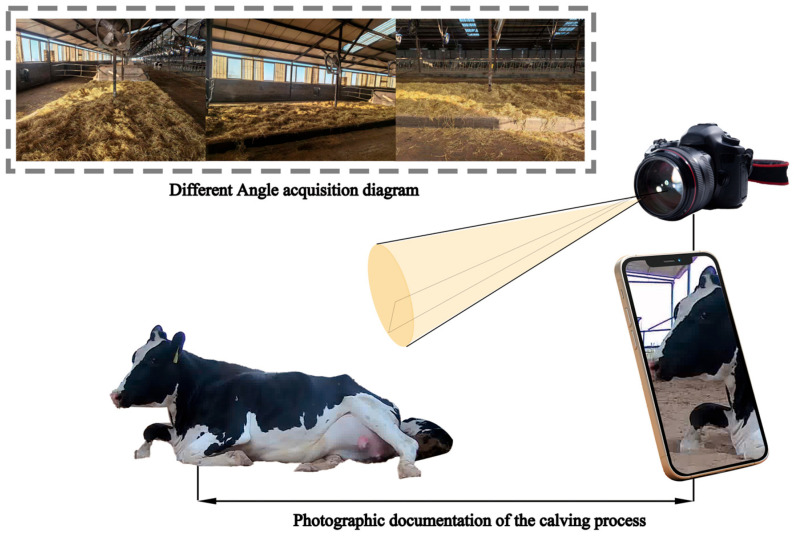
Schematic diagram of the collection site.

The experimental setup utilized computer vision-based detection tools, including cameras from Imou (IPC-G26E-IMOU) (Hangzhou Huacheng Network Technology Co., Ltd., Hangzhou, China) and Hikvision (DS-2CD3T46WD-I3) (Hangzhou Hikvision Digital Technology Co., Ltd., Hangzhou, China), along with a smartphone connected to Imou Cloud for real-time monitoring and a computer for video image processing. These devices provided full HD resolution (1920 × 1080) at up to 25 frames per second, with infrared capability for nighttime monitoring. Cameras were strategically mounted on iron posts or railings, adjustable to maintain a filming distance of approximately 3–4 m around the dedicated parturition shed, which measured 10 m × 10 m with additional 3 m × 10 m corridors on each side and a central parturition area (Figure 1). This setup ensured comprehensive coverage of the parturition process of cows nearing delivery, documented interference-free via smartphone. Captured videos (1920 × 1080) were edited to remove irrelevant footage and stored on a data storage hard drive.

### 2.2. Analysis of the Duration of Head Back During the Lying Parturition Process in Dairy Cows

All video frames were initially annotated by a single trained observer. Comprehensive video recordings of dairy cows during the parturition process, captured from an abdominal angle, were subjected to detailed frame-by-frame analysis at a rate of one frame per second. To evaluate inter-observer reliability, 10% of frames were randomly selected and independently labeled by a second observer blinded to the original annotations. Specifically, this dataset included 40 videos in total, evenly divided between primiparous and multiparous cows. Additionally, these videos were stratified by the neonatal weight of the calves, with 20 videos featuring calves weighing less than 37 kg and another 20 with calves weighing more than 43 kg. The videos encompassed the entire parturition process, from the initial appearance of the amniotic sac at the cow’s posterior to the complete expulsion of the calf. Quantitative image analysis was performed to classify each frame into one of three head posture categories: *T*_1_ (head back), *T*_2_ (no head back), and *T*_3_ (other postures). The *T*_3_ category was defined as any posture observed during parturition that did not meet the criteria for *T*_1_ or *T*_2_. This included all standing behaviors and clear transitions between lying and standing or between *T*_1_ and *T*_2_, but it excluded ambiguous or fleeting postural changes lasting less than two seconds. For each cow, the duration and frequency of occurrences of these postures were computed along with the total duration of all types (*T_t_*), where *T_t_* is the sum of *T*_1_, *T*_2_, and *T*_3_. For behavioral analysis at different stages of calving, the entire parturition period (from initial appearance of the amniotic sac to complete expulsion of the calf) was divided into three equal time intervals for each cow: early stage (first third), mid stage (second third), and late stage (final third). This standardized segmentation enabled a comparison of behavioral dynamics across cows with varying total parturition durations.

### 2.3. Construction of Dataset for Recognition Model

**Figure 2:**
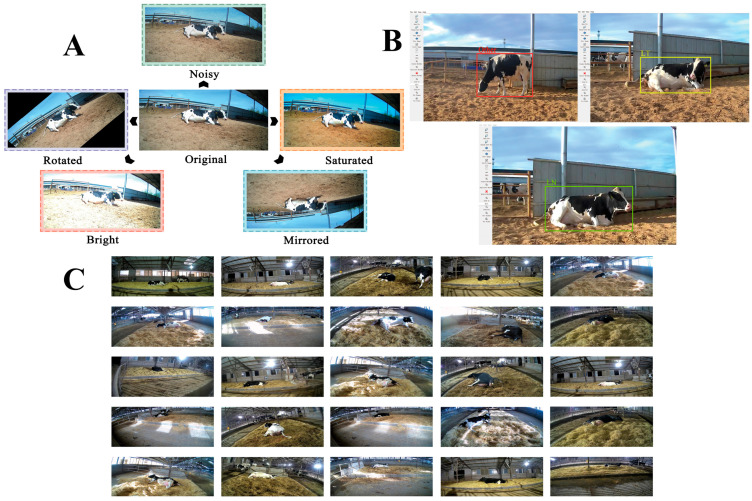
Construction of dataset for recognition model. (**A**) Schematic diagram of data enhancement. (**B**) Schematic diagram of representative annotations. (**C**) Head pose image dataset.

Video recordings were processed using Format Factory v5.21.0.0 software, with frames extracted at intervals of 50 frames (about every two seconds at 25 fps) and saved in JPG format. This setting captured adequate behavioral variation while minimizing redundancy. All extracted images underwent a manual screening process to exclude poor quality or irrelevant images—specifically those that were blurry, obstructed by equipment or other animals, poorly illuminated, or captured during camera transitions. To further enhance image robustness and improve model generalizability across varying conditions, we applied a series of data augmentation techniques to the remaining high-quality images. These included adjustments to saturation and brightness levels, addition of random noise, as well as geometric transformations such as rotation and vertical flipping (Figure 2A). For the purposes of annotation, the LabelImg tool was utilized within a Python3.8 environment.

Following that, images were categorized into three distinct states, lying without head back, lying with head back, and other (Table 1), and then divided into training, validation, and test sets in an 8:1:1 ratio at the individual cow level to prevent data leakage. There was no overlap of images or individuals between these subsets; each image belonged exclusively to one set. Representative annotations are illustrated in Figure 2B. Explicitly, the dataset comprised 8324 images of lying without head back, 12,083 images of lying with head back, and 5210 images categorized as other, totaling 25,617 images. Figure 2C presents a sample from the image dataset.

### 2.4. Training of the YOLOv8 Recognition Model

**Figure 3:**
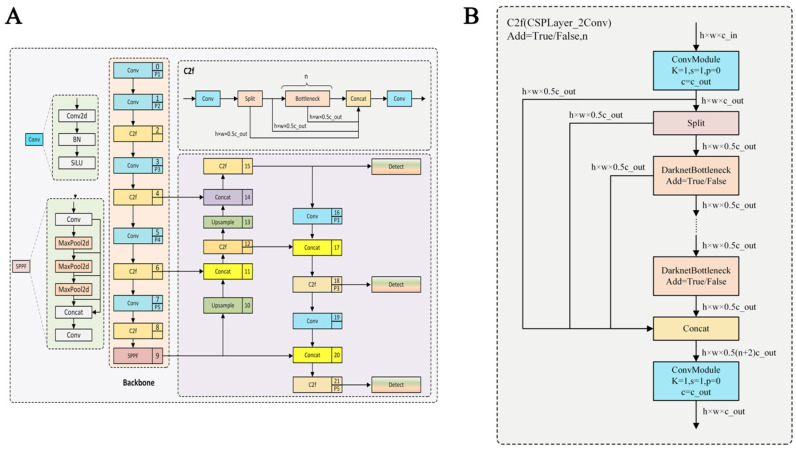
Training of the YOLOv8 recognition model. (**A**) Schematic diagram of the YOLOv8 framework. (**B**) Schematic diagram of the C2f network architecture.

YOLOv8, a deep learning-based object detection algorithm, was employed to recognize the head behaviors of dairy cows during parturition. For this purpose, the image dataset along with corresponding target detection TXT files were compiled into a dataset conforming to the VOC2007 standard, as utilized for training the YOLOv8 model (Figure 3A). Moreover, the C2f network architecture was chosen because it is integrated as the default backbone in YOLOv8 and is specifically designed to enhance feature extraction while maintaining computational efficiency. Its structure improves gradient flow and parameter utilization, making it effective for large-scale image datasets and complex object detection tasks such as behavioral monitoring in dairy cows (Figure 3B). The training involved annotating representative frames depicting various head behaviors of parturient dairy cows and conducting the training over 300 epochs, leveraging the YOLOv8 model. In this study, an “epoch” is defined as one complete pass through the entire training dataset during model training. Multiple epochs were used to iteratively update the model parameters and optimize performance.

The performance of the YOLOv8 model was quantitatively assessed applying precision (*P*), recall (*R*), average precision (*AP*), and *F*_1_ score, defined as follows:

(1) $$\;\;P=\frac{T_{p}}{T_{p}+F_{p}}\times 100\%\;\;$$

(2) $$R=\frac{T_{p}}{T_{p}+F_{n}}\times 100\%$$

(3) $$F1=\frac{2\times PR}{P+R}$$

(4) $$AP=\int_{0}^{1}p(r)dr$$

where *P*: the ratio of correctly classified positive instances to the total predicted as positive, *R*: the ratio of correctly classified positive instances to the total actual positive instances, true positives: the number of instances correctly predicted as positive, false positives: the number of instances incorrectly predicted as positive, false negatives: the number of actual positives incorrectly predicted as negative, *AP*: a method to summarize the precision–recall curve into a single value representing average precision across recall levels, and *F*_1_ score: the weighted average of *P* and *R*.

### 2.5. Model Validation

**Figure 4:**
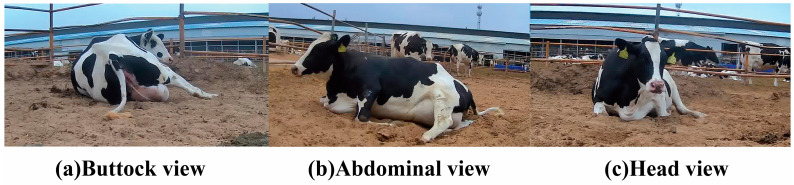
Schematic diagram of photographing angle. (**a**) Buttock view. (**b**) Abdominal view. (**c**) Head view.

For external validation, a separate dataset of 8961 images was assembled independently from the primary dataset used for model training. These images were collected from additional cows and video sequences that were not present in the main dataset, ensuring no overlap or data leakage. The validation set included approximately 1534 from buttock views, 1332 from head views, and 1395 images from abdominal views; it also covered various lighting conditions (1382 daytime, 1225 sunny, 1058 nighttime, and 1035 overcast) (Figure 4 and Table 2). The validation process employed the trained YOLOv8 model to recognize and assess the head postures of dairy cow during parturition. The primary evaluation metric used was *AP*, which provided a quantitative measure of the model’s recognition capabilities across different scenarios.

### 2.6. Model Training Environment and Parameter Settings

The experiments were conducted on a Windows platform using a machine equipped with an NVIDIA GeForce RTX 3050 GPU. Model development and training were performed using PyTorch 1.8, facilitated by CUDA 11.1 for efficient GPU acceleration (Table 3). The YOLOv8 implementation used in this study was version 8.0.0, which was the latest stable release available at the time of model development. We also measured the average inference time per image during model deployment. The detection speed was assessed by running the trained model on a randomly selected subset of test images and recording the time required to process each image. Due to computational resource limitations, an extensive grid or random search was not performed. Instead, preliminary runs were conducted using these defaults to confirm stable training convergence and adequate model performance on our dataset.

### 2.7. Data Processing and Analysis

The data of this study were analyzed using SPSS 19.0 software. Prior to applying parametric tests, we assessed normality using the Shapiro–Wilk test and verified homogeneity of variances using Levene’s test; all variables included in these analyses met these assumptions. The *t*-test was used to compare the differences in TP (time of parturition, min), CW (calf weight, kg), *T*_1_ (head back, min), *T*_2_ (no head back, min), and THB (times of head back, times) between first-time and experienced calving. One-way ANOVA was employed to compare differences in various indicators at different stages of primiparous and multiparous cows. If any variable had failed to meet these assumptions, an appropriate non-parametric alternative would have been considered; however, this was not necessary for our dataset. The charts were created using GraphPad Prism 10.1.2 and Origin 8.0 software.

## 3. Results

### 3.1. Characteristics of Head Back During the Lying Parturition Process in Dairy Cows

Head back is a primary behavioral feature observed during the parturition of dairy cows and is significantly influenced by the parity of the animal. Our findings demonstrated distinct differences in the duration and variability of this posture between primiparous and multiparous cows. For primiparous cows, the total duration of all types (*T_t_*) ranged from 18.62 to 109.00 min. The average durations were as follows: *T_t_* was 41.57 min; time spent in head back posture (*T*_1_) was 23.19 min; time spent in no head back posture (*T*_2_) was 11.99 min; and time spent in other postures (*T*_3_) was 6.40 min. By comparison, multiparous cows exhibited shorter total parturition durations, ranging from 10.77 to 58.00 min, and their average durations were as follows: total parturition time (*T_t_*) was 25.87 min; time spent in head back posture (*T*_1_) was 10.00 min; time spent in no head back posture (*T*_2_) was 9.82 min; and time spent in other postures (*T*_3_) was 6.05 min. These results indicated that primiparous cows not only maintain the head back posture for longer durations overall but also display greater variability in this duration across individuals. Further analysis of posture proportions revealed notable differences between the two parity groups. Primiparous cows spent the largest proportion of time in *T*_1_, accounting for 55.78% of their total duration, a value significantly higher than the 38.69% observed in multiparous cows (Table 4). These differences suggest that primiparous cows experience more prolonged and possibly less efficient parturition behaviors compared to their multiparous counterparts. The observed variability and prolonged head back posture in primiparous cows could be attributed to their lack of previous parturition experience, leading to differences in physical and behavioral adaptations during labor. In addition to parity, calf birth weight emerged as another critical factor influencing head back behavior during parturition. Cows delivering calves weighing less than 37 kg exhibited shorter durations across all posture categories. Specifically, the average durations for *T_t_*, *T*_1_, *T*_2_, and *T*_3_ were 26.49 min, 12.28 min, 9.32 min, and 4.89 min, respectively, with *T*_1_ being the most prolonged, accounting for 48.36% of the total duration. By contrast, cows with calf birth weights exceeding 43 kg exhibited average durations of 41.02 min, 23.95 min, 9.52 min, and 7.55 min for *T*_t_, *T*_1_, *T*_2_, and *T*_3_, respectively, where *T*_1_ similarly constituted the largest proportion at 58.39% of the total duration (Table 5). These findings underscore the significant impact of calf birth weight on maternal head back behavior during parturition, with a more pronounced head back posture observed in cows birthing heavier calves.

### 3.2. Evaluating the Performance of YOLOv8 in Recognizing Calving Cow Head Postures

**Figure 5:**
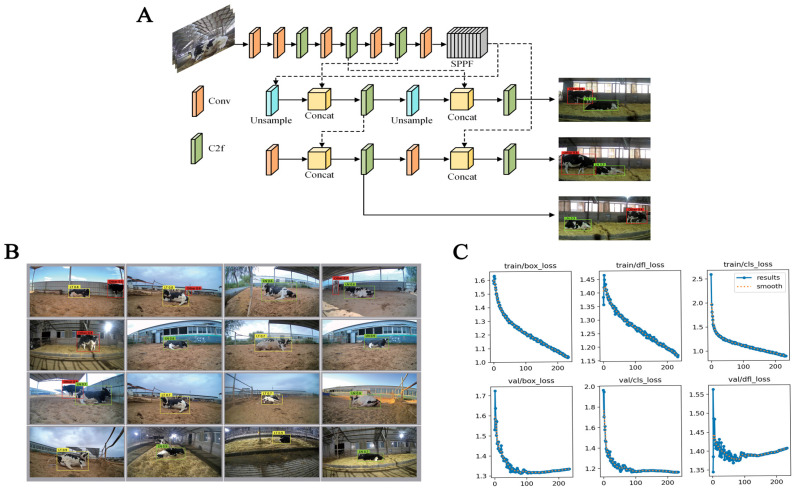
Overview of model architecture and performance. (**A**) Schematic of the recognition structure in YOLOv8 model. (**B**) Performance visualization of the YOLOv8 model. (**C**) Loss metrics for the YOLOv8 model as a function of epochs, depicting the model’s optimization progress over training cycles.

During the calving process, cows exhibit significant changes in head posture, with head back being a typical and easily identifiable pose. Leveraging this behavioral observation, we developed a recognition model for head back posture in calving cows leveraging the YOLOv8 algorithm (Figure 5A). The model was trained with images of calving cows exhibiting various head postures. After 233 epochs, the model automatically classified the images into three distinct states: lying without head back, lying with head back, and other postures (Figure 5B).

The performance metrics of the YOLOv8-based recognition model were as follows: *P* was 66.31%, *R* was 70.49%, *AP* was 68.0%, and *F*_1_ score was 0.68. These metrics indicated that the model achieved a moderate level of accuracy in distinguishing between different head postures, balancing detection rates with false positives and false negatives. While these results are promising for initial automated monitoring applications, they also highlight areas where further improvement is necessary. A more detailed breakdown of performance by posture category revealed that the *P* values for recognizing “lying without head back,” “lying with head back,” and “other” postures were 65.1%, 65.76%, and 68.1% respectively, while the *R* values were 68.71%, 75.35%, and 73.08% respectively (Table 6). These results demonstrated the model’s effectiveness in accurately detecting positive examples. The *F*_1_ score, which provides a balanced measure of *P* and *R*, reflected the model’s balanced detection performance, with all categories exceeding an *F*_1_ score of 0.65. The *AP* values, derived from the area under precision–recall curve, were around 70% for all behaviors, indicating a strong fit to the dataset and high predictive accuracy.

To further evaluate the model’s learning progression, we initialized training with a base of 300 epochs. The loss function exhibited a decreasing trend throughout the training process, converging around the 233rd epoch (Figure 5C). Beyond this point, the validation loss remained relatively stable, suggesting that the model had reached the limits of its learning capacity. Additional training beyond 233 epochs did not result in any significant performance improvement, suggesting that the model had achieved optimal training duration. This observation not only underscores the importance of monitoring loss trends during training but also highlights the model’s ability to generalize effectively without overfitting the dataset. In summary, these results confirmed that the model can effectively distinguish between lying and non-lying positions, enabling the exclusion of cows not in a lying down posture from analyses focused on calving-specific head movements.

### 3.3. Validation Analysis of Camera Angle and Environmental Impact on Recognizing Calving Cow Head Postures

Subsequent analysis revealed significant variation in the accuracy of posture recognition via camera angle (Table 7). The highest *AP* value was observed when using abdominal angle, with values exceeding 90% for all posture categories: lying without head back, lying with head back, and other postures. This superior performance was likely due to the abdominal angle’s ability to capture the full range of calving cow postures, providing a more comprehensive view of both head and body movements. This angle was particularly effective in capturing the amplitudes of head movements and changes in posture, which are critical for accurate classification. By contrast, capturing images from head angle yielded mixed results: 60.2% *AP* for lying without head back, a low 34.5% *AP* for lying with head back, and 70.3% *AP* for other postures. While the head angle provided a direct and detailed view of the head, it often failed to capture the full body, which was essential for recognizing posture-dependent head movements. This limited perspective likely reduced the model’s ability to discern subtle postural changes during calving, particularly for head back posture, where the body context plays a critical role in accurate recognition. The buttock angle proved to be the least effective, with *AP* values of 43.2%, 50.1%, and 75.2%. The reduced visibility of the head from this angle, due to obstruction by the cow’s body, likely hindered accurate detection of head movements and overall posture. Additionally, the buttock angle provided minimal information about the head and neck regions, which are crucial for differentiating between posture categories.

Further tests were conducted to assess the impact of environmental variations—specifically comparing sunny versus overcast conditions and daytime versus nighttime scenarios—on model performance employing the abdominal camera angle. The results indicated negligible differences in *AP* values, which remained above 90% across all conditions (Table 8). This robustness can likely be attributed to the large size of cows, which minimizes the impact of lighting variations. Coincidentally, even under challenging lighting conditions, such as low ambient light during nighttime or uneven illumination caused by shadows in sunny conditions, the model maintained high performance. This highlights the reliability of the abdominal angle for posture recognition across diverse environments, making it highly adaptable for real-world applications.

### 3.4. Analysis of Behavioral Changes in Primiparous and Multiparous Dairy Cows During Calving

**Figure 6:**
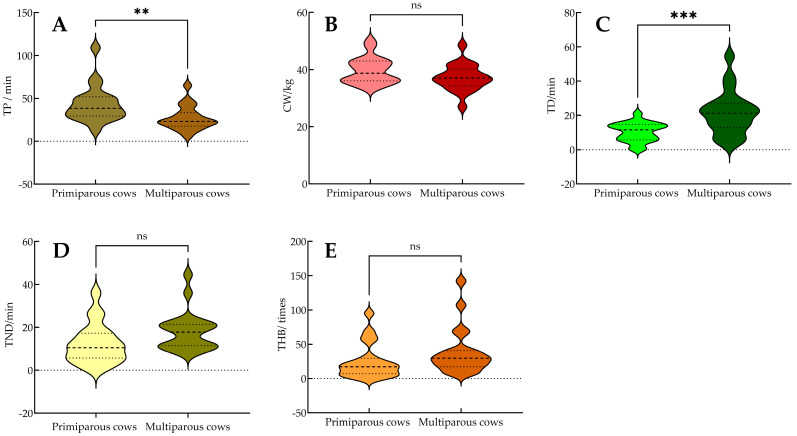
Comparative analysis of calving behavior durations in primiparous and multiparous dairy cows. (**A**–**E**) Differences in time of parturition (TP, min), calf weight (CW, kg), time of head back (TD, min), time of no head back (TND, min), and times of head back (THB, times) between primiparous and multiparous dairy cows. Note: The surface of each violin plot represents the distribution density of the data for each stage, while different colors are used to distinguish among behavioral variables and cow groups. ** indicates a significant difference within the same group (*p* < 0.01), *** indicates a significant difference within the same group (*p* < 0.001), while “ns” denotes a non-significant difference within the group (*p* > 0.05).

In this study, changes in the TP, CW, TD, TND, and THB values of primiparous cows and multiparous cows were explored. The results showed that the TP values and the TD values of primiparous cows were significantly higher than those of multiparous cows (*p* < 0.05), while there was no significant difference in CW, TND, and THB values between primiparous cows and multiparous cows (Figure 6A–E). Among the 40 cows covered in this study, the maximum TP value (109 min) appeared in primiparous cows, and the minimum TP value (6 min) appeared in multiparous cows. The average TP values of primiparous cows and multiparous cows were 43.1 min and 26.85 min, respectively, and the average total head back time was 21.53 min and 10.30 min, respectively. The above data show that the delivery duration of primiparous cows is longer, and turning back is the main activity during delivery.

**Figure 7:**
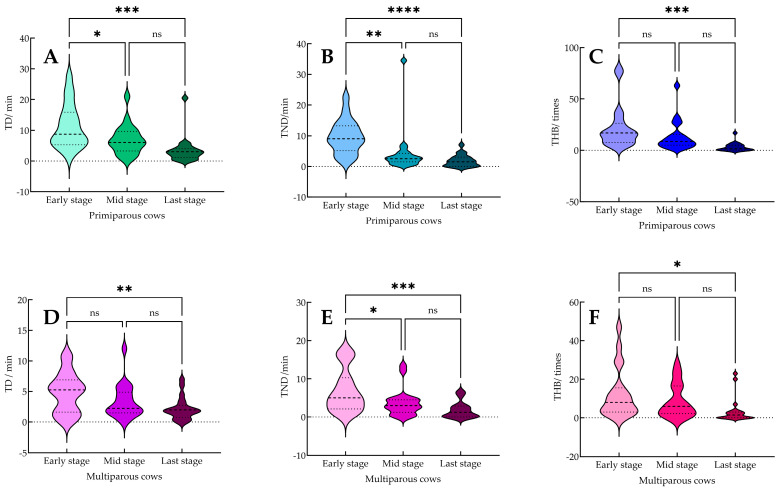
Comparative analysis of the duration of calving behavior between primiparous and multiparous cows at different stages of delivery. (**A**–**C**) Variations in TD, TND, and THB across different stages of calving in primiparous cows, and (**D**–**F**) corresponding variations in multiparous cows. Note: The surface of each violin plot represents the distribution density of the data for each stage, while different colors are used to distinguish among behavioral variables and cow groups. * indicates a significant difference within the same group (*p* < 0.05), ** indicates a significant difference within the same group (*p* < 0.01), *** indicates a significant difference within the same group (*p* < 0.001), **** indicates a significant difference within the same group (*p* < 0.0001), while “ns” denotes a non-significant difference within the group (*p* > 0.05). “Early stage,” “Mid stage,” and “Last stage” represent the different phases of the calving process.

There are significant differences in the length of delivery of dairy cows between different parities and individuals, and the dynamic characteristics of movements during delivery show obvious differences. In this study, the performance of primiparous cows and multiparous cows at different stages of delivery was evaluated. The results showed that both groups exhibited decreasing trends in TP, TD, TND, and THB from early to late stages. While durations were generally longer in the early stage, differences in frequency and intensity of head back behavior were also observed between stages and parities. It is worth noting that there were differences in TD, TND and THB in the early stage of primiparous cows compared with the late stage (*p* < 0.05) (Figure 7A–C). Compared with the middle stage, there was a difference between TD and TND (*p* < 0.05); compared with the late stage, there was only a difference in TND (*p* < 0.05). The early and late stages of multiparous cows showed similar patterns (Figure 7D–F). Compared with the early, middle, and late stages, only TND was different in the early and middle stages (*p* < 0.05), and there was no difference in TD and THB (*p* > 0.05). These findings confirmed that *T*_1_ and *T*_2_ movements are most prominent in the early stage of parturition. In addition, the TD, TND, and THB values in the early stage of primiparous cows were 3.27, 4.96, and 7.11 times higher than those of multiparous cows, respectively, which indicated that compared with multiparous cows, primiparous cows had more frequent turning back, longer duration of turning back, and higher frequency of turning back in the early stage of delivery.

### 3.5. Correlation Between Maternal Behavior Patterns During Primiparous and Multiparous Calving in Dairy Cows

**Figure 8:**
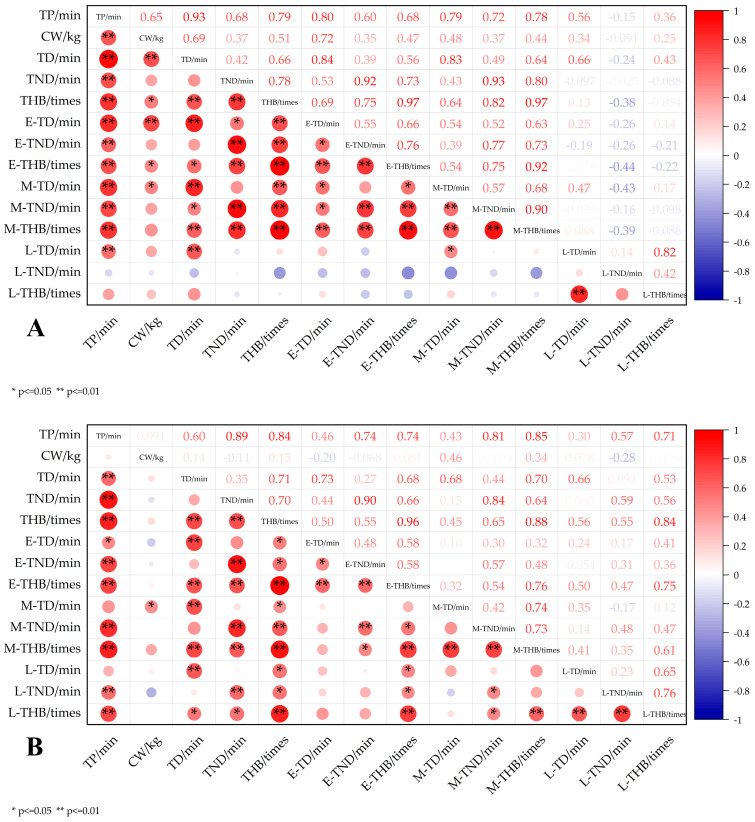
Comparative analysis of calving behavior durations in (**A**) primiparous and (**B**) multiparous dairy cows. TP: time of parturition (min), CW: calf weight (kg), TD: time of head back (min), TND: time of no head back (min), THB: times of head back (times), E-TD: time of head back during early stage (min), E-TND: time of no head back during early stage (min), E-THB: times of head back during early stage (times), M-TD: time of head back during mid stage (min), M-TND: time of no head back during mid stage (min), M-THB: times of head back during mid stage (times), L-TD: time of head back during last stage (min), L-TND: time of no head back during last stage (min), L-THB: times of head back during last stage (times).

In this study, the duration index during delivery was calculated, and Pearson correlation analysis was used to explore the correlation between TP, TD, TND, THB, and CW during and after delivery. During the process of primiparity, there was a significant positive correlation between the length of TD in different stages (Figure 8A), such as TD in the early and middle stages (*p* < 0.05, r = 0.54). The duration of TND also showed a strong correlation in each stage, with no looking back in the early and middle stages (*p* < 0.01, r = 0.77). In addition, there was a high positive correlation between THB in each stage, early and middle (*p* < 0.01, r = 0.916).

The correlation analysis of multiparous cows showed that TP was significantly positively correlated with TD (*p* < 0.01, r = 0.60), TND (*p* < 0.01, r = 0.89), and THB (*p* < 0.01, r = 0.84) (Figure 8B). At different stages, the duration of the pre-delivery period was significantly correlated with head movement, indicating that this stage had a significant effect on the cow’s head movement. In the middle of delivery, there was a high positive correlation with THB and TD, indicating that the duration of this stage had a greater impact. The correlation between CW and TD was generally weak, and most correlations were not significant, except for the significant correlation between mid-post head up (*p* < 0.05, r = 0.46), indicating that CW during multiparity had little effect on the T_1_ of cows at different stages. It is important to note that while some correlations were statistically significant due to sample size, only coefficients above 0.5 were considered to reflect moderate or strong biological relationships. There was a certain positive correlation between TD of the head in each stage, and the correlation with TND was strong. THB in the early, middle, and late stages was highly positively correlated, indicating that the behaviors of multiparous cows in different stages were consistent.

Through the comparative analysis of the correlation between primiparous and multiparous cows, the correlation between TP and CW was stronger in primiparous cows and weaker in multiparous cows, indicating that there were differences in the delivery mechanism between primiparous and multiparous cows. Primiparous cows may be more sensitive to CW, while the effect of CW on TP is reduced due to adaptive changes in body function of multiparous cows. The correlation between TP and TND was higher in multiparous cows than in primiparous cows, indicating that TND was more affected by TP during the delivery of multiparous cows, which may be due to changes in behavioral patterns after multiple deliveries. In general, the CW at primiparity was strongly correlated with other indicators, while it was generally weak at multiparity, which further indicated that there were significant differences in the physiological and behavioral responses of cows during primiparity and multiparity. Therefore, we can determine the state of the cow through the early stage of delivery, enabling manual intervention to be carried out in advance to ensure the smooth delivery process.

## 4. Discussion

The periparturient period is marked by a high incidence of morbidity and severe mortality rates, with approximately 30% to 50% of dairy cows affected by some form of postpartum disease, leading to substantial resource wastage and economic losses on farms [22]. Studies have indicated that cows delivering stillborn calves exhibit more pre-partum activity than those delivering live calves, suggesting elevated pain levels [23]. This observation underscores the importance of monitoring parturition behaviors to provide timely assistance during calving. Effective monitoring can significantly prevent neonatal losses and promote animal welfare by enabling early intervention strategies that address complications promptly [24,25,26,27]. The head back posture is considered a natural mechanism by which dairy cows cope with the pain of parturition. By altering head position, cows may distract themselves and alleviate pain, potentially triggering the endogenous analgesia system, such as the release of endorphins, thus aiding them in better enduring the parturition process [28]. The frequency and duration of head back posture could reflect the progress of parturition, as this behavior tends to increase as labor advances [14]. Consequently, this behavior can serve as a non-invasive indicator to assess the progress of parturition, assisting farm managers in determining the need for intervention [29]. Our study highlights significant behavioral differences between primiparous and multiparous cows during calving, suggesting variability in pain sensitivity, coping capacity, and overall health status. The more prolonged head back posture observed in primiparous cows may be attributed not only to physical factors—such as less-developed pelvic tissues, narrower birth canals, or greater soft tissue resistance—but also to psychological aspects like increased anxiety or unfamiliarity with the calving process. By contrast, multiparous cows benefit from previous exposure to calving, which may result in physiological adaptations such as improved uterine contractility and cervical dilation, as well as behavioral acclimatization. These adaptive changes can facilitate a shorter and less stressful parturition process. Indeed, compared to multiparous cows, primiparous cows exhibit higher step counts [11,30] and more total lying time [11], with most studies confirming that first-time calving takes longer and typically involves greater effort than subsequent calvings. Moreover, the duration of head back posture correlates with neonatal weight, providing insights into the physical challenges cows face during parturition. Calves born weighing over 43 kg are associated with longer and more intense ‘head back’ posture, implying a more strenuous parturition process. This is likely due to heavier calves causing longer and more complicated deliveries, where the significant weight at birth markedly impacts the ease of parturition, often necessitating more assistance [31]. This relationship underscores the importance of monitoring and potentially offering assistance during calving involving heavier calves to prevent complications and promote maternal and neonatal health [32]. Thus, by observing and quantifying head back behavior, valuable information can be gleaned about the welfare status of dairy cows, aiding in the development of personalized care strategies.

YOLOv8 is an advanced object detection model based on deep learning that is highly regarded for its speed and accuracy in real-time detection tasks [33]. In the medical field, YOLOv8 enhances the diagnostic process through its ability to rapidly and accurately detect anatomical features and pathological markers within medical imaging [34]. In the livestock industry, YOLOv8 has made substantial advancements in monitoring and managing livestock [35,36,37]. In this study, we developed a model using the YOLOv8 algorithm that classifies images into three distinct postures: lying without head back, lying with head back, and other. With a dataset of 25,617 images, the model demonstrated effective performance in posture recognition. The deployment of the YOLOv8 algorithm in our study underscores its robustness in real-time object detection. The model’s *P*, *R*, *AP*, and *F*_1_ score collectively indicated a competent distinction capacity between these postures. The *F*_1_ score, in particular, is a crucial metric for measuring model accuracy, especially in classification tasks. It integrates *P* and *R* to assess the efficiency of the model in detecting specific objects [38]. A high *F*_1_ score indicates that the model achieves an optimal balance between minimizing false positives and false negatives, which is vital for decision making and interventions in practical applications. The *F*_1_ scores—0.67, 0.71, and 0.69 for the three categories, respectively—indicated that the model achieves a balanced yet moderate level of accuracy in detecting specific behaviors. While these results demonstrate the system’s potential for supporting behavioral monitoring on dairy farms, it should be acknowledged that this level of performance cannot yet be considered fully robust or ready for widespread deployment without further refinement. From a practical standpoint, an *F*_1_ score of 0.71 for ‘lying with head back’ indicates that approximately 71% of actual head back events were correctly identified by the system while maintaining a balanced rate of false positives and false negatives. For farmers using this technology in real-time monitoring scenarios, this means that most true cases requiring attention (e.g., potential signs of pain or dystocia) would be flagged promptly by the system; however, some events might still be missed or incorrectly triggered due to ambiguous postures or environmental interference. Optimizing camera placement can further improve detection rates in practice. Ultimately, even with current performance levels, automated detection offers substantial advantages over manual observation by enabling continuous surveillance and rapid intervention when abnormal behaviors are detected.

The application of the YOLOv8 object detection algorithm in analyzing the postures of dairy cows provides crucial insights into the optimal camera angles and environmental conditions that influence the accuracy of behavioral recognition. This validation analysis, which examines the impacts of camera placement and environmental factors, is vital for optimizing the setup of automated monitoring systems in livestock management. It is well known that the angle from which the camera captures the animal is crucial for the effectiveness of posture recognition. The abdominal angle offers a comprehensive perspective of the cow’s body, enabling an exceptionally high *AP* of over 90% across all posture categories. This angle captures the full range of motion during the calving process, which is essential for accurate behavioral analysis. By contrast, head and buttock angles demonstrate significantly lower *AP* due to their limited viewpoints, which obscure essential parts of the body that are crucial for precise posture recognition. In addition, environmental factors such as lighting conditions also impact the accuracy of posture recognition. The study findings indicate that, regardless of daytime or nighttime, the substantial size of dairy cows and standardized lighting facilitate the robustness of the YOLOv8 model, maintaining high *AP* across varying conditions. This resilience against environmental changes benefits continuous monitoring without the need for frequent system adjustments. By selecting optimal camera angles and considering environmental factors, farmers and veterinarians can significantly enhance the accuracy and reliability of automated systems in identifying key events during calving, thereby monitoring and managing animal welfare.

Despite these findings, it must be acknowledged that our study has several limitations. It is well known that larger datasets significantly enhance the accuracy and generalizability of models. In this study, we included a total of 25,617 images, comprising 8324 images of lying without the head back, 12,083 images of lying with the head back, and 5210 images categorized as other. However, future work should involve training our model with even larger datasets to further improve its performance. One limitation of this study is the absence of ablation studies, as no modifications were made to the original YOLOv8 architecture. Should future research involve enhancements or additional modules, systematic ablation analyses will be conducted. Additionally, our research demonstrates that variations in camera angles substantially affect the recognition outcomes. Specifically, the recognition performance was highest at the abdominal angle, with *AP* values exceeding 90% across all posture categories: lying without the head back, lying with the head back, and other postures. However, the practical implementation of imaging devices at optimal angles in production environments remains a challenge and requires further optimization. Another limitation concerns the generalizability of our findings: all data were collected from Holstein cows at a single commercial dairy farm under standardized management conditions. While this approach minimized confounding variables within our study population, it may limit the applicability of our model to other breeds or farms with different housing systems, management practices, or environmental conditions. Future research should validate the YOLOv8-based detection system across diverse herds and operational settings to confirm its robustness and adaptability. Finally, the implementation of an automated computer vision-based calving monitoring system requires an initial investment in hardware—primarily high-resolution cameras capable of functioning under various lighting conditions, as well as computers with sufficient processing power (preferably with GPU acceleration) for real-time image analysis. Open-source software platforms such as PyTorch 1.8 can minimize software costs; however, technical expertise is needed for model deployment and maintenance. Additional costs may include data storage solutions for large volumes of video footage and periodic staff training to ensure optimal operation of the system. While these investments are higher than those associated with traditional manual observation, they are offset by potential long-term savings in labor costs, improved detection of calving-related problems, enhanced animal welfare, and reduced economic losses from dystocia or delayed intervention.

## 5. Conclusions

This study demonstrates that a YOLOv8-based computer vision model can effectively and non-invasively monitor the “head back” posture in dairy cows, providing valuable real-time insights into calving behavior. Our results show that both parity and calf birth weight significantly affect labor duration and head back posture, and that optimal camera placement—particularly at the abdominal angle—greatly improves detection accuracy. While the system offers clear advantages for continuous monitoring and timely intervention, further work is needed to expand dataset diversity, validate performance across different breeds and farm environments, and refine model components. Overall, automated behavioral monitoring holds strong promise for advancing animal welfare and calving management in modern dairy farming.

## Figures and Tables

**Table 1:** Definition of head posture in parturient cows.

Target Categories	Definitions
Lying without head back	During parturition, the dairy cow is typically prone with the head naturally oriented forward
Lying with head back	During parturition, the dairy cow is in a recumbent position with the head turned backward, exhibiting a head back posture
Other	During parturition, the dairy cow does not exhibit normal recumbent parturition postures, showing standing or transitional standing behavior

**Table 2:** Composition of the validation dataset.

Items	Validation Set Size	Collection Locations	Collection Time
Buttock	1534	Barns 1–4	July, August, and September
Head	1332		
Abdomen	1395		
Sunny	1382		
Overcast	1058		
Daytime	1225		
Nighttime	1035		

**Table 3:** Model parameter settings.

Parameters	Number
epoch	300
input_shape	[600, 600]
anchors_size	[8, 16, 32]
momentum	0.9
weight_decay	0
save_period	5

**Table 4:** Duration of head postures during parturition in primiparous and multiparous cows.

	Primiparous Cows (n = 20)					Multiparous Cows (n = 20)				
	*T*_1_ (min)	*T*_2_ (min)	*T*_3_ (min)	*T*_t_ (min)	Assisted Calving	*T*_1_ (min)	*T*_2_ (min)	*T*_3_ (min)	*T*_t_ (min)	Assisted Calving
1	6.93	19.67	12.65	39.25	No	1.2	18.47	4.67	24.33	No
2	1.8	15.68	1.13	18.62	No	2.4	5.7	2.67	10.77	No
3	8.78	29.77	0	38.55	No	8.75	11.45	3.4	23.6	No
4	16.27	3.28	0	19.55	No	8.8	12.63	0	21.43	No
5	15.6	3.9	0	19.5	No	7.22	8.68	0	15.9	No
6	4.27	15.73	0	20	No	3	27	0	30	No
7	8.78	10.65	0	19.43	No	7.15	5.07	6.05	18.27	No
8	26.53	21.9	0.98	49.42	Yes	2.98	7.68	5.22	15.88	No
9	31.7	0	8.62	40.32	Yes	4.32	19.5	0.5	24.32	No
10	14.17	4.22	15.32	33.7	No	10.02	6.08	0	16.1	No
11	25.78	26.87	0.98	53.63	Yes	2.1	5.23	4.33	11.67	No
12	28.45	12.97	7.03	48.45	No	12.68	12.32	0	25	No
13	53.17	8.67	47.17	109	Yes	18.07	6.93	35	60	No
14	33.87	17.13	0	51	Yes	16.25	5.85	10.9	33	No
15	29.9	7.73	7.37	45	No	16.63	10.2	11.17	38	No
16	21.05	3.63	3.32	28	No	20.85	9.8	27.35	58	Yes
17	49	3.48	7.52	60	Yes	14.9	2.1	4	21	Yes
18	28.02	12.38	4.6	45	No	12.98	4.67	5.35	23	No
19	28.33	16.67	0	45	No	14.77	6.97	0.27	22	No
20	31.33	5.4	11.27	48	Yes	15.07	9.93	0	25	Yes
Average duration ± standard deviation	23.19 ± 13.85	11.99 ± 8.38	6.4 ± 10.76	41.57 ± 20.55		10 ± 6.08	9.82 ± 5.98	6.05 ± 9.33	25.87 ± 13.12	
Proportion	55.78%	28.83%	15.39%	100%		38.69%	37.94%	23.37%	100.00%	

Note: Average duration is the average time of delivery of primiparous and multiparous cows, and Proportion is the proportion of *T*_1_, *T*_2_, and *T*_3_ to *T_t_* of primiparous and multiparous cows. Note: *T*_1_: head back, *T*_2_: no head back, and *T*_3_: other postures. The *T*_3_ category was defined as any posture observed during parturition that did not meet the criteria for *T*_1_ or *T*_2_. This included all standing behaviors and clear transitions between lying and standing or between *T*_1_ and *T*_2_, but it excluded ambiguous or fleeting postural changes lasting less than two seconds.

**Table 5:** Duration of head postures during parturition in cows with different calf birth weights.

	Calf Birth Weight Below 37 kg (n = 20)					Calf Birth Weight Above 43 kg (n = 20)				
	*T*_1_ (min)	*T*_2_ (min)	*T*_3_ (min)	*T*_t_ (min)	Assisted Calving	*T*_1_ (min)	*T*_2_ (min)	*T*_3_(min)	*T*_t_ (min)	Assisted Calving
1	19.67	6.93	12.65	39.25	No	16.63	10.2	11.17	38	No
2	15.68	1.8	1.13	18.62	No	20.85	9.8	27.35	58	Yes
3	29.77	8.78	0	38.55	No	14.9	2.1	4	21	Yes
4	4.22	14.17	15.32	33.7	No	12.98	4.67	5.35	23	No
5	3.9	15.6	0	19.5	No	8.8	12.63	0	21.43	No
6	15.73	4.27	0	20	No	15.07	9.93	0	25	Yes
7	10.65	8.78	0	19.43	No	16.27	3.28	0	19.55	No
8	18.47	2.87	4.67	26	No	26.53	21.9	0.98	49.42	Yes
9	6.93	18.07	35	60	No	31.7	0	8.62	40.32	Yes
10	16.67	28.33	0	45	No	25.78	26.87	0.98	53.63	Yes
11	5.7	2.4	2.67	10.77	No	28.45	12.97	7.03	48.45	No
12	11.45	8.75	3.4	23.6	No	53.17	8.67	47.17	109	Yes
13	8.68	7.22	0	15.9	No	33.87	17.13	0	51	Yes
14	27	3	0	30	No	29.9	7.73	7.37	45	No
15	5.07	7.15	6.05	18.27	No	21.05	3.63	3.32	28	No
16	7.68	2.98	5.22	15.88	No	31.33	5.4	11.27	48	Yes
17	19.5	4.32	0.5	24.32	No	2.1	5.23	4.33	11.67	No
18	6.08	10.02	0	16.1	No	12.68	12.32	0	25	No
19	5.85	16.25	10.9	33	No	49	3.48	7.52	60	Yes
20	6.97	14.77	0.27	22	No	28.02	12.38	4.6	45	No
Average duration ± standard deviation	12.28 ± 7.66	9.32 ± 6.77	4.89 ± 8.48	26.49 ± 12.06		23.95 ± 12.61	9.52 ± 6.76	7.55 ± 11.28	41.02 ± 21.59	
Proportion	46.36%	35.19%	18.45%	100%		58.39%	23.30%	18.41%	100%	

Note: Average duration is the average time of delivery of primiparous and multiparous cows, and Proportion is the proportion of *T*_1_, *T*_2_ and *T*_3_ to *T_t_* of primiparous and multiparous cows. Note: *T*_1_: head back, *T*_2_: no head back, and *T*_3_: other postures. The *T*_3_ category was defined as any posture observed during parturition that did not meet the criteria for *T*_1_ or *T*_2_. This included all standing behaviors and clear transitions between lying and standing or between *T*_1_ and *T*_2_, but it excluded ambiguous or fleeting postural changes lasting less than two seconds.

**Table 6:** Results of calving cow head posture recognition using the YOLOv8 algorithm.

Indicator	Model	Lying Without Head Back	Lying with Head Back	Other
Precision (*P*)	66.31%	65.10%	69.76%	68.47%
Recall (*R*)	70.49%	68.71%	75.35%	73.08%
Average Precision (*AP*)	68.03%	69.13%	70.12%	64.14%
*F*_1_ score	0.68	0.67	0.71	0.69

Note: Precision (*P*): the ratio of correctly predicted positive observations to the total predicted positive observations (true positives/[true positives + false positives]). Recall (*R*): the ratio of correctly predicted positive observations to all actual positive observations (true positives/[true positives + false negatives]). Average Precision (*AP*): a summary metric that combines precision and recall across different thresholds, representing the area under the precision-recall curve. *F*_1_ Score: the harmonic mean of precision and recall, providing a single score that balances both metrics.

**Table 7:** Validation results of model across different angles.

Items	Average Precision (*AP*)			Frame per Second (FPS)
	Lying Without Head Back	Lying with Head Back	Other	
Buttock (n = 1534)	43.20%	50.10%	75.20%	87
Head (n = 1332)	60.20%	34.50%	70.30%	76
Abdomen (n = 1395)	93.30%	92.50%	95.20%	83

**Table 8:** Validation results of the model in various environments.

Items	Average Precision (*AP*)			Frame per Second (FPS)
	Lying Without Head Back	Lying with Head Back	Other	
Sunny (n = 1382)	90.20%	94.20%	92.40%	73
Overcast (n = 1058)	90.40%	93.30%	94.50%	78
Daytime (n = 1225)	92.50%	96.30%	94.30%	82
Nighttime (n = 1035)	91.40%	94.30%	95.10%	79

## Data Availability

The original contributions presented in this study are included in the article. Further inquiries can be directed to the corresponding author.

## References

[1] Mee J.F. (2008). Prevalence and risk factors for dystocia in dairy cattle: A review. Vet. J..

[2] Keceli A.S., Catal C., Kaya A., Tekinerdogan B. (2020). Development of a recurrent neural networks-based calving prediction model using activity and behavioral data. Comput. Electron. Agric..

[3] Umaña Sedó S.G., Renaud D.L., Morrison J., Pearl D.L., Mee J.F., Winder C.B. (2024). Using an automated tail movement sensor device to predict calving time in dairy cows. JDS Commun..

[4] Lanzoni L., Chincarini M., Giammarco M., Fusaro I., Iannotta M., Podaliri M., Contri A., Gloria A., Vignola G. (2022). Changes in the behaviour before normal calving to predict its onset in Mediterranean buffaloes heifers. Appl. Anim. Behav. Sci..

[5] Higaki S., Koyama K., Sasaki Y., Abe K., Honkawa K., Horii Y., Minamino T., Mikurino Y., Okada H., Miwakeichi F. (2020). Technical note: Calving prediction in dairy cattle based on continuous measurements of ventral tail base skin temperature using supervised machine learning. J. Dairy Sci..

[6] Mainau E., Manteca X. (2011). Pain and discomfort caused by parturition in cows and sows. Appl. Anim. Behav. Sci..

[7] Ouellet V., Vasseur E., Heuwieser W., Burfeind O., Maldague X., Charbonneau É. (2016). Evaluation of calving indicators measured by automated monitoring devices to predict the onset of calving in Holstein dairy cows. J. Dairy Sci..

[8] Kebede A., Mohammed A.K., Tadessse W., Abera D., Nekemte E. (2017). Review on Economic Impacts of Dystocia in Dairy Farm and Its Management and Prevention Methods. Nat. Sci..

[9] Kovács L., Kézér F.L., Ruff F., Szenci O. (2016). Timing of obstetrical assistance affects peripartal cardiac autonomic function and early maternal behavior of dairy cows. Physiol. Behav..

[10] Kovács L., Kézér F.L., Szenci O. (2016). Effect of calving process on the outcomes of delivery and postpartum health of dairy cows with unassisted and assisted calvings. J. Dairy Sci..

[11] Edwards E.M., Krawczel P.D., Dann H.M., Schneider L.G., Whitlock B., Proudfoot K.L. (2020). Calving location preference and changes in lying and exploratory behavior of preparturient dairy cattle with access to pasture. J. Dairy Sci..

[12] Lamanna M., Bovo M., Cavallini D. (2025). Wearable Collar Technologies for Dairy Cows: A Systematized Review of the Current Applications and Future Innovations in Precision Livestock Farming. Animals.

[13] Cavallini D., Giammarco M., Buonaiuto G., Vignola G., De Matos Vettori J., Lamanna M., Prasinou P., Colleluori R., Formigoni A., Fusaro I. (2025). Two years of precision livestock management: Harnessing ear tag device behavioral data for pregnancy detection in free-range dairy cattle on silage/hay-mix ration. Front. Anim. Sci..

[14] Barraclough R.A.C., Shaw D.J., Boyce R., Haskell M.J., Macrae A.I. (2020). The behavior of dairy cattle in late gestation: Effects of parity and dystocia. J. Dairy Sci..

[15] Crociati M., Sylla L., De Vincenzi A., Stradaioli G., Monaci M. (2022). How to Predict Parturition in Cattle? A Literature Review of Automatic Devices and Technologies for Remote Monitoring and Calving Prediction. Animals.

[16] Ono Y., Hatano R., Ohwada H., Nishiyama H. (2019). Predicting Cow’s Delivery Using Movement and Position Data Based on Machine Learning. EasyChair.

[17] Szenci O. (2022). Accuracy to Predict the Onset of Calving in Dairy Farms by Using Different Precision Livestock Farming Devices. Animals.

[18] Saint-Dizier M., Chastant-Maillard S. (2018). Potential of connected devices to optimize cattle reproduction. Theriogenology.

[19] Saint-Dizier M., Chastant-Maillard S. (2015). Methods and on-farm devices to predict calving time in cattle. Vet. J..

[20] Sumi K., Zin T.T., Kobayashi I., Horii Y. A study on cow monitoring system for calving process. Proceedings of the 2017 IEEE 6th Global Conference on Consumer Electronics (GCCE).

[21] González-Sánchez C., Sánchez-Brizuela G., Cisnal A., Fraile J.C., Pérez-Turiel J., Fuente-López E. (2021). Prediction of Cow Calving in Extensive Livestock Using a New Neck-Mounted Sensorized Wearable Device: A Pilot Study. Sensors.

[22] Wisnieski L., Norby B., Pierce S.J., Becker T., Gandy J.C., Sordillo L.M. (2019). Predictive models for early lactation diseases in transition dairy cattle at dry-off. Prev. Vet. Med..

[23] Liboreiro D.N., Machado K.S., Silva P.R., Maturana M.M., Nishimura T.K., Brandão A.P., Endres M.I., Chebel R.C. (2015). Characterization of peripartum rumination and activity of cows diagnosed with metabolic and uterine diseases. J. Dairy Sci..

[24] Mao A., Huang E., Wang X., Liu K. (2023). Deep learning-based animal activity recognition with wearable sensors: Overview, challenges, and future directions. Comput. Electron. Agric..

[25] Eerdekens A., Deruyck M., Fontaine J., Martens L., De Poorter E., Plets D., Joseph W. (2021). A framework for energy-efficient equine activity recognition with leg accelerometers. Comput. Electron. Agric..

[26] Belaid M.A., Rodriguez-Prado M., López-Suárez M., Rodríguez-Prado D.V., Calsamiglia S. (2021). Prepartum behavior changes in dry Holstein cows at risk of postpartum diseases. J. Dairy Sci..

[27] Yang L., Zhao J., Ying X., Lu C., Zhou X., Gao Y., Wang L., Liu H., Song H. (2024). Utilization of deep learning models to predict calving time in dairy cattle from tail acceleration data. Comput. Electron. Agric..

[28] Proudfoot K.L., Jensen M.B., Weary D.M., von Keyserlingk M.A. (2014). Dairy cows seek isolation at calving and when ill. J. Dairy Sci..

[29] Borchers M.R., Chang Y.M., Tsai I.C., Wadsworth B.A., Bewley J.M. (2016). A validation of technologies monitoring dairy cow feeding, ruminating, and lying behaviors. J. Dairy Sci..

[30] Mainau E., Cuevas A., Ruiz-de-la-Torre J.L., Abbeloos E., Manteca X. (2014). Effect of meloxicam administration after calving on milk production, acute phase proteins, and behavior in dairy cows. J. Vet. Behav..

[31] Streyl D., Sauter-Louis C., Braunert A., Lange D., Weber F., Zerbe H. (2011). Establishment of a standard operating procedure for predicting the time of calving in cattle. J. Vet. Sci..

[32] von Keyserlingk M.A., Rushen J., de Passillé A.M., Weary D.M. (2009). Invited review: The welfare of dairy cattle—Key concepts and the role of science. J. Dairy Sci..

[33] Jiang P., Ergu D., Liu F., Cai Y., Ma B. (2022). A Review of Yolo Algorithm Developments. Procedia Comput. Sci..

[34] Clark P.M., Raggatt P.R., Price C.P. (1985). Antibodies interfering in immunometric assays. Clin. Chem..

[35] Saifudin A.L.I., Madyawati S.P., Yuadi I., Rimayanti R., Mustofa I., Rulaningtyas R., Gunawan T.S., Besari A.R.A. (2024). Monitoring cow behavior based on lying, standing, eating, and ruminating recognition using YOLOv8. Turk. J. Vet. Anim. Sci..

[36] Zhao K., Duan Y., Chen J., Li Q., Hong X., Zhang R., Wang M. (2023). Detection of Respiratory Rate of Dairy Cows Based on Infrared Thermography and Deep Learning. Agriculture.

[37] Peng Y., Peng Z., Zou H., Liu M., Hu R., Xiao J., Liao H., Yang Y., Huo L., Wang Z. (2024). A dynamic individual method for yak heifer live body weight estimation using the YOLOv8 network and body parameter detection algorithm. J. Dairy Sci..

[38] Yu Z., Liu Y., Yu S., Wang R., Song Z., Yan Y., Li F., Wang Z., Tian F. (2022). Automatic Detection Method of Dairy Cow Feeding Behaviour Based on YOLO Improved Model and Edge Computing. Sensors.

